# Influence of Neoadjuvant Chemotherapy on Survival Outcomes of Radical Cystectomy in Pathologically Proven Positive and Negative Lymph Nodes

**DOI:** 10.3390/cancers15194901

**Published:** 2023-10-09

**Authors:** Krystian Kaczmarek, Bartosz Małkiewicz, Karolina Skonieczna-Żydecka, Artur Lemiński

**Affiliations:** 1 Department of Urology and Urological Oncology, Pomeranian Medical University, Powstańców Wielkopolskich 72, 70-111 Szczecin, Poland; 2 University Center of Excellence in Urology, Department of Minimally Invasive and Robotic Urology, Wroclaw Medical University, Borowska 213, 50-556 Wroclaw, Poland; 3 Department of Biochemical Sciences, Pomeranian Medical University, Władysława Broniewskiego 24, 71-460 Szczecin, Poland

**Keywords:** neoadjuvant chemotherapy, radical cystectomy, occult nodal disease, bladder cancer, overall survival

## Abstract

**Simple Summary:**

Understanding how effective a treatment is before a major bladder surgery can help doctors to plan better patient care. Our research investigated the survival rates of patients who received neoadjuvant chemotherapy before having their bladders removed due to cancer. We wanted to examine if there were differences in survival for those who had certain signs of cancer in their lymph nodes after the chemotherapy. Our findings suggest that those who showed these signs and had received prior chemotherapy had a more challenging health outlook than those who directly went for surgery. This information is crucial as it may guide doctors to consider additional treatments and closer patient monitoring in certain cases. Our study helps to provide a clearer picture for both doctors and patients when making decisions about bladder cancer treatment.

**Abstract:**

Patients receiving neoadjuvant chemotherapy (NAC) prior to radical cystectomy (RC) typically show better survival outcomes than those undergoing immediate surgery for muscle-invasive bladder cancer. However, most studies have not considered the lymph node (LN) status when evaluating NAC’s survival benefits. This study sought to delineate the impact of NAC on patients based on their pathologically determined LN status at the time of RC. We examined data from 1395 patients treated at two departments between 1991 and 2022. Of them, 481 had positive LNs. A comparison of overall survival (OS) outcomes revealed that patients without LN involvement ((y)pN0) benefited from NAC with a hazard ratio (HR) of 0.692 (95% confidence interval [CI] 0.524–0.915). In contrast, patients with (y)pN+ showed no improvement with NAC (HR 0.927, 95%CI 0.713–1.205). Notably, patients treated with NAC for stage <ypT2ypN+ tumours experienced reduced OS compared to their counterparts who did not receive NAC. The HR was 3.111 (95%CI 1.249–7.746). Given that persistent nodal disease after NAC correlates with a worse prognosis, additional post-operative treatments should be considered.

## 1. Introduction

Neoadjuvant chemotherapy (NAC) stands as the gold standard in the multidisciplinary treatment of muscle-invasive bladder cancer (MIBC). All patients eligible for cisplatin combination chemotherapy should be considered for NAC before undergoing a radical cystectomy (RC) or trimodal therapy (TMT). Among the NAC regimens, dose-dense methotrexate, vinblastine, doxorubicin, and cisplatin (ddMVAC) regimens are viewed the most potent in neoadjuvant contexts. Undergoing NAC often leads to a positive pathological response. Notably, approximately 40–50% of patients are downstaged to ypT0ypN0, and 20–30% to ≤ypT1pN0 [1,2]. The tangible benefits of NAC, manifesting in improved tumour control, account for a 5% uptick in the 5-year overall survival (OS) and a 9% increment in the 5-year disease free survival (DFS) when contrasted with RC alone [3]. Moreover, studies have demonstrated that NAC can also reduce sex-related disparities in oncological results, with women showing marked responses, especially those initially diagnosed with extravesical disease [4]. The most suitable candidates for NAC are those with cT2–4 tumours without any radiological indications of a node-positive disease. Whereas the most promising NAC outcomes are observed in patients diagnosed with pure urothelial carcinoma and MIBC with small cell neuroendocrine variants [5]. For such patients, the risk of detecting positive lymph nodes (LNs) in the RC specimen is markedly reduced. Nevertheless, a subset of patients exhibits occult nodal disease despite the guidelines for NAC. Currently, the medical community lacks a unanimous guideline or definitive recommendation concerning the management of patients with ypN+. Furthermore, data on the survival outcomes of patients who are clinically negative but have pathologically confirmed disease post-NAC are scarce. While we can infer the prognostic significance of NAC for these patients from the results of induction chemotherapy in clinically positive LN patients who still show nodal disease after systemic therapy. However, given their poor prognosis, many cN+ patients only undergo induction chemotherapy. Notably, responses to systemic therapy differ; recent studies have revealed that roughly 50% of patients with cN+ show pathologically positive LNs, a rate substantially higher than that in patients with no presurgical imaging-based LN suspicions [6]. Additionally, the discrepancy in responses to systemic therapy between patients with the cN0 and cN+ disease was noticeable at the local tumour stage. The ypT0 stage rate was significantly lower in patients with cN+. Zargar-Shoshtari et la. reported only a 15% local response rate in patients with cN+ [7]. Some researchers have argued that the count of excised LNs significantly influences the long-term oncological outcomes in patients with cN+ treated with induction chemotherapy [7,8], whereas the merits of extended pelvic lymph node dissection (PLND) remain debated for patients treated with NAC [9]. Consequently, the oncological outcomes for patients with pathologically verified LNs post-induction chemotherapy may not mirror those post-NAC. Therefore, this study aimed to determine the role of NAC in patients with clinically negative, but pathologically verified positive LNs.

## 2. Materials and Methods

We conducted a non-randomized clinical follow-up study in accordance with the Declaration of Helsinki. The Institutional Review Board (Bioethical Committee) of the Pomeranian Medical University, Szczecin, Poland, exempted this research from further review. All participating patients provided consent for the utilization of their anonymized treatment data collected during their hospitalization. Our study comprised consecutive patients who underwent RC and PLND for MIBC at two university centres: the University Centre of Excellence in Urology of Wroclaw Medical University, Poland, and the Department of Urology and Urological Oncology of the Pomeranian Medical University, Szczecin. All treatments spanned from 1991 to 2022. We excluded patients from further analysis if they were diagnosed with metastatic disease. The presence of metastases was evaluated through thoracic and abdominopelvic computed tomography (CT) taken pre-RC and post-NAC when applicable. The clinical LN stage was determined based on factors including size (≥8 mm in short-axis diameter), shape, and internal features like necrosis or a typical fatty hilum [10]. We further excluded patients who underwent cystectomy for palliative reasons, such as haematuria or chronic pain; received a partial cystectomy; had a previous history of pelvic radiotherapy; and presented with non-urothelial pathology. Finally, 112 patients were excluded from the study, leaving data from 1394 patients for statistical analyses. 

During the RC, PLND followed a standardized template that covered the obturator, internal, external, and common iliac LN up to the crossing of the ureter. However, in certain instances, the extent of PLND was adjusted based on the surgeon’s intraoperative judgment. Extended PLND included LNs situated above the common iliac bifurcation [11]. In terms of multidisciplinary treatment at the Department of Urology and Urological Oncology of the Pomeranian Medical University, Szczecin, NAC was being consistently offered to patients with MIBC qualified to RC since 2017, and the uptake of NAC has been progressively increasing in the following years. Therefore, the Galsky criteria were widely adopted in this department [12], whereas at the Department of Minimally Invasive and Robotic Urology, University Center of Excellence in Urology of Wroclaw Medical University, NAC has been routinely considered in patients before RC since 2002. Hence, the administration of NAC at this department before 2012 is based on criteria presented in Nordic Cystectomy Trial 2 [13]. In terms of cisplatin-based chemotherapy, patients on the GC regimen received gemcitabine 1000 mg/m^2^ on days 1, 8, and 15 plus cisplatin 70 mg/m^2^ on day 2 [14], whereas patients on the MVAC regimen received methotrexate 30 mg/m^2^ on days 1, 15, and 22; vinblastine 3 mg/m^2^ on days 2, 15, and 22; doxorubicin 30 mg/m^2^ on day 2; and cisplatin 70 mg/m^2^ on day 2 [15]. Cycles were repeated every 28 days. Patients received a maximum of six cycles of treatment. The choice between both cisplatin-based regimens was made by the oncological team. Considering similar efficacy of both regimens, the results from a randomized phase 3 trial have also been used to justify the routine use of GC regimen in patients with more comorbidities [14]. Since 2014, when a promising result for the dose-dense fashion of an MVAC regimen was presented in a prospective phase II study, this regimen has been considered as a standard of care for MIBC in the neoadjuvant setting [16]. Patients on the ddMVAC regimen received methotrexate 30 mg/m^2^ on day 1 and vinblastine 3 mg/m^2^, doxorubicin 30 mg/m^2^, and cisplatin 70 mg/m^2^ on day 2. If patients did not meet cisplatin-based criteria, alternative regimens within the NAC framework were explored. Specifically, carboplatin-based chemotherapy was recommended for patients with an ECOG PS of at least 2 and a GFR range of 30–60 mL/min. However, if the GFR was below 60 mL/min, ECOG PS was less than 2, and the patient had adequate bone marrow reserves, taxane-based chemotherapy was considered after a comprehensive evaluation by the multidisciplinary team. Notably, no patient in this study received immune-oncology therapy prior to RC. The pathological complete response (pCR) to NAC was defined as reaching the ypT0pN0 stage, whereas the pathological partial response (pPR) was noted if patients achieved the ypTis/pTa/pT1pN0 stage. Patients with persistent MIBC or who had progression after NAC administration were categorized as no responders. 

**Figure 1:**
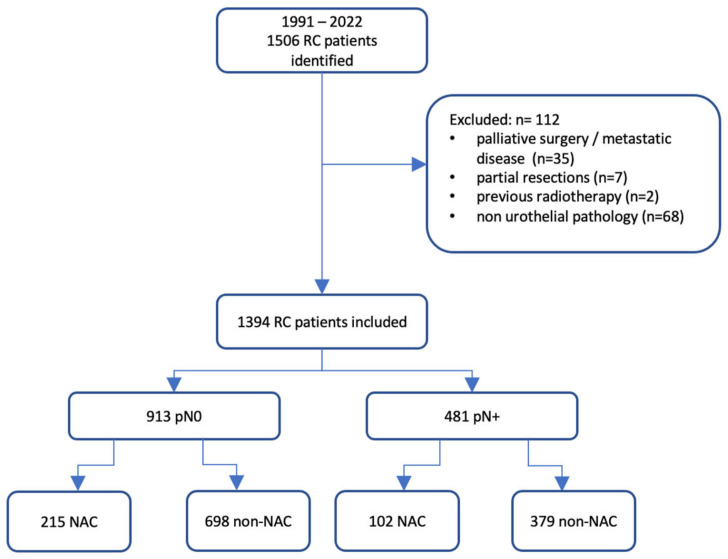
Flowchart of the study. NAC: neoadjuvant chemotherapy; RC: radical cystectomy.

The analysed cohort was divided into two groups according to pathological LN status in RC specimens. The first group comprised patients with positive LNs, while the control group consisted of those with confirmed negative LNs in the pathological assessment. To discern variations between patients who were or were not subjected to NAC, we investigated the correlation between NAC and overall survival (OS) in both groups, aligned with their pathological LN stage (refer to Figure 1). OS was demarcated as the span from the RC date either to the date of death or the last recorded follow-up, with no restriction on the cause of death.

### Statistical Analysis

Data were reviewed for internal consistency by two authors (K.K. and A.L.). Normally distributed reviewed data were characterized using mean and standard deviation, whereas medians accompanied by interquartile ranges described skewed data. Differences between the (y)pN0 and (y)pN+ groups were discerned using independent *t*-tests for parametric variables and chi-square tests for nonparametric variables. Kaplan–Meier survival estimates along with univariate Cox analysis were employed to depict OS probabilities across time. The log-rank test facilitated the comparison of survival curves. Multivariate Cox proportional hazard models were used to assess the impact of prognostic factors on survival, including age at the time of surgery, sex, severity of comorbidities as reflected by the American Society of Anaesthesiologists (ASA) score, pathological T stage, pathological N stage, cancer grade, and NAC administration in each analysed group. The multivariate Cox regression analyses pertaining to the predictors of OS were conducted for the entire cohort and for each group separately. Independence between residuals and time, essential for verifying the proportional hazard assumption of the concluding multivariable models, was examined using scaled Schoenfeld residuals. Results of the multivariate Cox regression analyses are presented as hazard ratios (HRs) and 95% confidence intervals (CIs). Considering the non-homogeneity of the analysed groups, a propensity scores based on the noted variables were determined. A logistic regression was used to calculate these propensity scores, predicting the likelihood of pathologically positive LN post-RC. The patients in the (y)pN0 bracket were matched with those in the (y)pN+ bracket, followed by a multivariate propensity-weighted Cox regression analysis. The threshold for statistical significance was established at 0.05, with all *p*-values being two-sided. Analytical procedures were executed using Statistica software (version 13.5) (StatSoft, Inc., Tulsa, OK, USA), R (version 4.2.2) and RStudio (version 2022.12.0) with the R packages *survival*, *survminer*, and *drylr*.

## 3. Results

Of the 1395 patients included in the final analysis, 481 (34.51%) presented with positive LNs, while the control group included 913 (65.49%) individuals. The average follow-up duration for these groups was 23.56 months and 41.42 months, respectively. No remarkable differences in terms of age or sex were discerned between the LN-positive and LN-negative groups. Nevertheless, an uneven distribution was observed for the ASA scores, with a larger percentage of pN+ patients having an ASA score of ≥3, compared with pN0 patients (39.71% vs. 32.86%; *p* = 0.011). Additionally, significant disparities arose in tumour characteristics. Patients in the pN+ category exhibited invasive disease more frequently, as per clinical and pathological evaluations, than those in the control group. High-grade tumours were more commonly diagnosed in the pN + cohort. The selection of NAC regimen showed no variance in the pN0 and pN+ groups. Nevertheless, a higher fraction of individuals in the pN+ category had been administered a subpar number of NAC cycles. Specifically, 43.14% (44/102) of the patients in the pN+ group, who received NAC prior to RC, underwent fewer than three chemotherapy cycles. Conversely, in the pN0 group, only 27.44% (59/215) of patients were given a suboptimal cycle count (*p* = 0.005). The demographic and clinical characteristics of the study population are summarized in Table 1.

Overall, 317 (22.74%) patients received NAC. The most common preoperative regimen was ddMVAC, administered to 53.31% of these patients. The pCR and pPR in NAC pretreated patients were achieved in 18.29% and 16.72%, respectively. In comparison, those undergoing RC without prior NAC had lower rates of downstaging: pCR at 5.66% and pPR at 13.65% (*p* < 0.001). Notably, persistent LN disease was observed in a similar proportion of patients in the NAC (32.18%) and non-NAC (35.19%) groups (*p* = 0.321). Among those with persistent nodal disease, the NAC group had a higher incidence of non-muscle bladder cancer (NMIBC) in RC specimens at 23.53%, compared with 6.07% in the non-NAC groups (*p* < 0.001).

**Figure 2:**
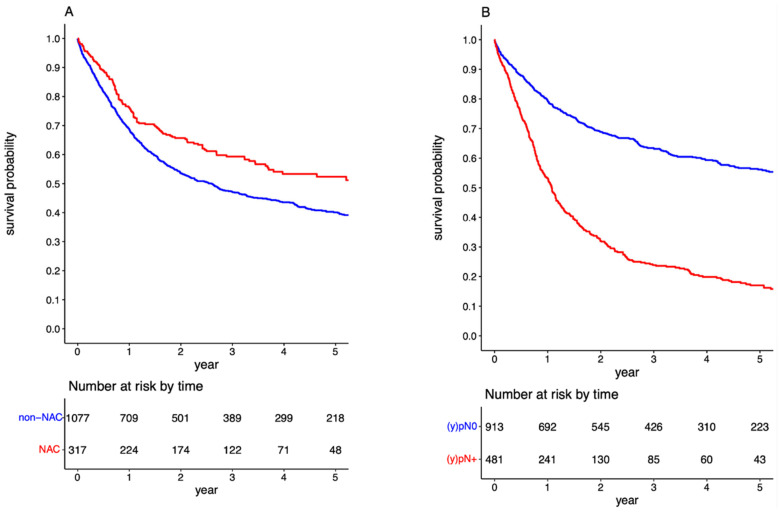
Survival curves for overall survival in the overall cohort stratified by administration of neoadjuvant chemotherapy (**A**); and by pathologic lymph node status (**B**).

The OS at 3 and 5 years for the entire cohort was 49.84% (95%CI: 47.09–52.60) and 42.69% (95%CI: 39.78–45.59), respectively. Patients with negative LNs exhibited notably better survival rates post-RC, with 3-year and 5-year OS at 63.27% (95%CI: 60.00–66.53) and 56.16% (95%CI: 52.55–59.76), respectively. This was contrastingly higher than the LN-positive population, who had a 3-year and 5-year OS of 23.88% (95%CI: 19.79–27.96) and 17.01% (95%CI: 13.17–20.85), respectively, with a significant *p*-value of <0.001 (Figure 2). The detrimental effect of pathologically confirmed positive LNs on long-term survival was further substantiated through a multivariable Cox regression analysis, yielding an HR of 2.075 (95%CI: 1.780–2.419; Table S1). When evaluating the influence of NAC, it was discerned that patients who received preoperative systemic therapy demonstrated significantly enhanced OS compared to NAC than those who directly underwent RC (*p* < 0.001). Specifically, in the non-NAC group, the 3-year and 5-year OS were 47.15% (95%CI: 44.04–50.26) and 40.11% (95%CI: 36.89–43.33). Conversely, for those pre-treated with NAC, the 3-year and 5-year OS were 59.33% (95%CI: 53.56–65.10) and 52.39% (95%CI: 45.94–58.84), respectively (refer to Figure 2). This survival advantage associated with NAC was further corroborated using a multivariable Cox regression analysis, presenting an HR of 0.807 (95%CI: 0.667–0.977; Table S1).

**Figure 3:**
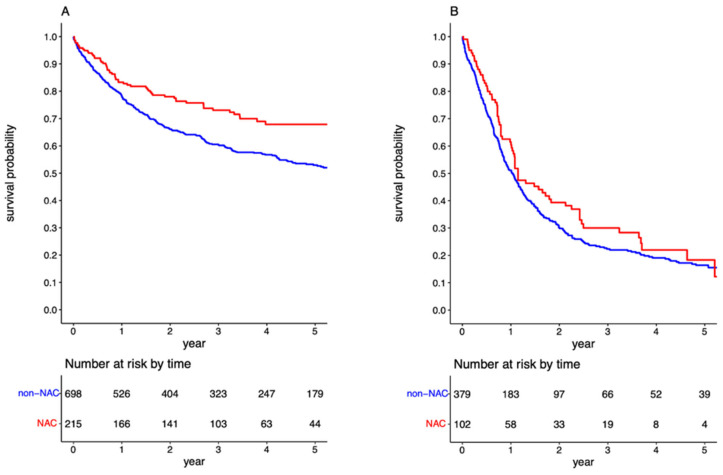
Survival curves for overall survival in the overall cohort stratified by administration neoadjuvant chemotherapy in the (y)pN0 (**A**) and (y)pN+ cohorts (**B**).

Following cohort stratification based on the LN status, the survival benefit of NAC was evident in the (y)pN0 group (*p* < 0.001; Figure 3A). In contrast, there was no further improvement in prognosis for the (y)pN+ group administered NAC (*p* = 0.123, Figure 3B). The 5-year OS for (y)pN0 patients exposed and not exposed to NAC was 67.86% (95%CI: 60.74–74.98) and 52.97% (95%CI: 48.88–57.05), respectively, with an associated HR of 0.692 (95%CI: 0.524–0.915). The propensity-weighted analysis yielded an HR of 0.714 (95%CI: 0.608–0.838). Among patients with positive LNs, the 5-year OS for those exposed and not exposed to NAC was 18.31% (95%CI: 7.77–28.85) and 16.80% (95%CI: 12.62–20.98), respectively. The HR for pre-surgical chemotherapy in the (y)pN+ cohort was 0.927 (95%CI: 0.713–1.205).

**Figure 4:**
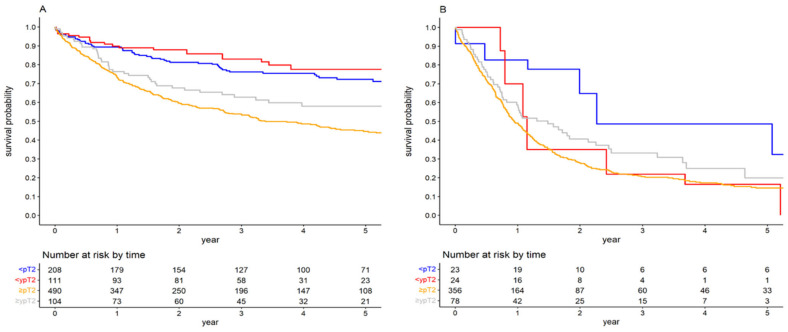
Survival curves for overall survival stratified by local tumour stage in the (y)pN0 (**A**) and (y)pN+ cohorts (**B**).

Further stratification based on the local downstaging in RC specimens revealed that in patients with negative LNs, the notable impact of NAC on OS persisted across local tumour stages. The 5-year OS for patients with negative LNs and local tumour stage <pT2, <ypT2, ≥pT2, and ≥ypT2 were 72.22% (95%CI: 65.61–78.83), 77.49% (95%CI: 68.21–86.79), 44.58% (95%CI: 39.66–49.50) and 57.98% (95%CI: 47.53–68.42), respectively (*p* < 0.001, Figure 4A). The HR for NAC in the <(y)pT2pN0 cohort was 0.572 (95%CI: 0.343–0.951), while for the ≥(y)pT2pN0 cohort, it was 0.694 (95%CI: 0.494–0.975). Nonetheless, the propensity-weighted analysis did not indicate any NAC effect in the <(y)pT2pN0 group (HR 0.791; 95%CI: 0.584–1.072). In contrast, patients with positive LNs exhibited differing outcomes. For the (y)pN+ cohort achieving the <(y)pT2 stage, NAC appeared to be detrimental in terms of OS. The 5-year OS rates for <(y)pT2pN+ cohorts with and without NAC were 16.41% (95%CI: 0.68–32.14) and 48.59% (95%CI: 23.19–74.00), respectively, (Figure 4B). The associated HR was 3.111 (95%CI: 1.249–7.746). The difference in OS for <(y)pT2pN+ cohorts was also pronounced after the exclusion of patients who received a suboptimal number of cycles (HR 2.361, 95%CI: 1.034–5.395). For the ≥(y)pT2pN+ cohort, NAC did not exhibit any pronounced effect on OS (HR 0.752; 95%CI: 0.556–1.016). The 5-year OS rates for ≥(y)pT2pN+ cohorts with and without NAC were 19.92% (95%CI: 6.98–32.88) and 14.47% (95%CI: 10.48–18.48), respectively. Comprehensive results from the multivariate Cox regression models are shown in Table 2, whereas the propensity-weighted analysis outcomes can be found in Supplementary Material (Table S2). 

## 4. Discussion

A response to NAC after debulking transurethral resection of bladder tumours has been noted in up to 50% of patients [2]. This therapeutic response was specifically defined by the achievement of downstaging to ypT0pN0 or ≤ypT1pN0 post-RC. The transition from NAC to RC is not immediate, requiring interim evaluations to assess the potential for bladder-sparing treatments. This re-evaluation phase entails comprehensive abdominopelvic CT scanning combined with a detailed cystoscopic analysis of the bladder. This, in conjunction with a resection of the primary bladder cancer site, is vital. The absence of LN metastasis and downstaging to <ypT2 paves the way for the possible implementation of trimodal therapy (TMT) [17]. The decision between opting for the RC and TMT is a shared decision. It is crucial that patients are fully informed of the advantages and disadvantages tied to each therapeutic approach. For those considering bladder preservation, it is paramount that the bladder does not exhibit unfavourable characteristics, such as the presence of multifocal cT2 tumours, lesions >5 cm, or concurrent carcinoma in situ. Moreover, patients should also have a well-functioning bladder with an adequate capacity and no hydronephrosis [18,19,20]. Then, the selected patients may undergo radical chemoradiotherapy (CRT). Different radiosensitising chemotherapy regimens were used in a radical CRT setting. However, due to the lack of randomized trials and limited evidence regarding the most effective radiosensitizers, the combined administration of mitomycin C and 5-fluorouracil with either cisplatin or gemcitabine appears to be a reasonable option [21,22,23]. Regarding RT protocols, a multiplicity of recommendations exists. In many North American trials, the initial RT dose typically ranges from 39.6 to 45 Gy, targeting the pelvic LNs situated below the bifurcation of the common iliac vessels, the entire bladder, and, in males, the prostate [24]. However, attempts have been made to improve bladder preservation rates while minimising therapy toxicity. Consequently, the idea of excluding pelvic LN from the initial radiation treatment has garnered considerable attention. This approach, by reducing the volume of normal tissue exposed, is believed to improve therapy tolerability. Nonetheless, given the underlying risk of occult nodal disease in patients with the <ypT2 disease, incorporation of the pelvic LN in the early stages of the radiation field seems logical. Of course, beyond only achieving local downstaging, NAC’s imperative function is to address the potential micrometastatic spread of bladder cancer. Therefore, current trends show an inclination towards forgoing whole pelvic radiation in TMT, with some advocating for the omission of extended PLND during RC. In a focused trial, Tunio et al. found no significant disparities in survival metrics, bladder preservation rates, or regional nodal failure outcomes between patient cohorts subjected to whole pelvic radiation versus bladder-specific radiation using the TMT approach [25]. Furthermore, Lemiński et al. suggested that patients pre-treated with NAC did not experience enhanced survival benefits from adequate PLND [26]. Contrarily, our findings identified occult nodal disease post-NAC in patients who opted for RC with PLND over TMT. The incidence of LN disease was documented at 12.12% for the patients downstaged to ypT0 and 23.19% for those at the ypT1 stage. A significant majority of ypN+ patients (66.67%) were administered the GC regimen as a part of their NAC. Moreover, one third of these are provided with fewer than the optimal cycle count. The frequency of occult nodal disease after NAC has yielded varied data. Mertens et al. associated heightened LN involvement post-NAC with initially diagnosed extravesical disease, pinpointing an incidence rate of up to 20% for patients with occult nodal micrometastases following preoperative chemotherapy [27]. More promising results were reported by van Hoogstraten et al. In their study, only 4.3% of the patients who achieved the <ypT2 stage after neoadjuvant therapy had occult nodal disease [6]. However, this study population exhibited primary extravesical disease in only 19.8% of cases. This contrasts with our cohort, where 78.8% presented with cT3-T4a disease. It is evident why a pronounced rate of occult nodal disease was observed in our study.

Post-RC, patients showing a local response to <(y)pT2 disease with confirmed pathologically positive LN emerged as a distinctive patient subgroup. The existing literature offering insights on the long-term prognosis of this category is sparse. In our research, patients with the <ypT2pN+ disease demonstrated inferior survival outcomes compared with those with the <pT2pN+ disease. The 5-year OS for <ypT2N+ was quantified at 16.41% with a 95%CI spanning 0.68–32.14, whereas for <pT2N+, it stood at 48.59% with a 95%CI of 23.19–74.00 and an associated HR of 3.111 backed by a 95%CI of 1.249–7.746. Ploussard et al. presented similar findings, underscoring that residual, nodal disease in RC specimens post-NAC correlated with a reduced OS than pN1–3 disease succeeding primary RC. It is pivotal to note that their analysis did not delineate based on the localized tumour stage [28]. Certain studies have detailed the outcomes of patients with ypN+ disease, relying on a solitary observational study model without juxtaposing with the non-NAC group. A limited-scope study by Jeong et al. reported on 53 patients, all subjected to NAC followed by either partial or RC, and unequivocally presented with pathologically confirmed LN metastases. The recorded 2-year recurrence-free survival (RFS) and OS rates were 23.3% and 34.6%, respectively [29]. Owing to limited data regarding the different prognoses of patients with <ypT2N+ and <pT2N+ disease, we believe that our study provides notable findings in this field. Our results indicate that despite achieving local downstaging, the presence of a chemoresistant pathology can substantially worsen a patient’s prognosis. Such insights are imperative when TMT is under consideration. Occult nodal disease may substantially deteriorate the TMT outcomes, particularly when RT is singularly directed at the bladder. Future research initiatives should aspire to craft nomograms to pre-emptively detect occult nodal disease in patients after NAC administration. Such nomograms would be instrumental in a shared decision-making process, particularly when all the treatment options after NAC are listed in a table. 

To date, definitive guidelines regarding the treatment of patients with positive LN post-NAC remain absent. The option of introducing adjuvant chemotherapy (AC) exists; however, due to the inherent chemoresistance, its efficacy is compromised. Furthermore, results regarding the use of AC after NAC usually came from single-arm retrospective studies, encompassing patients with locally advanced diseases (ypT3/4) without LN metastasis. Additionally, the literature on this subject is fragmented, often presenting incongruent findings. For instance, preliminary data by Seisen et al. suggest that integrating AC post-NAC and RC may improve the OS in patients with pT3/T4 and/or pN+ disease [30]. Conversely, Zargar-Shoshtari et al. found that conventional AC did not substantially improve RFS or cancer-specific survival [31]. Other research documented bladder cancer recurrence during AC administration [29]. Moreover, the effectiveness of AC against persistent nodal disease seems questionable, regardless of NAC’s presence. Data from the EORTC 30994 trial revealed disparities in the AC outcomes based on the LN status. Notably, only patients without nodal metastasis seemed to benefit from AC in terms of OS [32]. Consequently, owing to the lack of firm evidence, the current guidelines do not recommend AC for patients with ypN+. In addition to oncological results, the feasibility of AC is another important factor, which must be considered in this group of patients. Donat et al. demonstrated that up to 30% of the patients undergoing RC are unable to undergo AC because of the postoperative complications [33]. As an alternative, adjuvant radiotherapy (ART) emerges as a potential intervention for patients with ypN+. Research by Lewis et al. assessed ART’s ramifications post-NAC and RC, revealing that ART is primarily beneficial for patients with positive surgical margins [34]. Given the subpar outcomes associated with both AC and ART in the ypN+ demographic, novel therapeutic agents are under examination. Notably, the CheckMate 274 trial probed the adjuvant application of nivolumab. Their findings underscored nivolumab’s potential in enhancing DFS, particularly among NAC-treated patients with confirmed nodal disease [35]. In this study, we did not analyse the effect of adjuvant treatment in the ypN+ cohort. We were unable to perform this analysis because of a lack of data regarding adjuvant treatment in a larger proportion of the patients. Nevertheless, we believe that such treatment might yield favourable survival outcomes, especially within the <ypT2N+ subgroup. 

While our analysis offers intriguing insights, there are inherent limitations to the study that require consideration. Primarily, the observational nature introduces potential biases that must be acknowledged when interpreting the results. Specifically, we were unable to measure certain confounding variables, including socioeconomic status, comorbidities using the Charlson Comorbidity Index, smoking status, and the time between the end of NAC and RC and the initiation of adjuvant chemotherapy. These factors could significantly influence survival outcomes. Consequently, our analysis does not definitively determine whether a postoperative systemic treatment would be beneficial for patients with LN positivity following NAC and RC. Further complicating our analysis is the sporadic availability of cause of death data. This limitation necessitates a focus on OS in our survival analysis, which may introduce additional biases owing to potential overlapping causes of mortality within the cohort. Also, it is important to note the novel therapeutic opportunities in neoadjuvant settings in MIBC. The preliminary data from ongoing studies signal promising outcomes from new agents, such as checkpoint inhibitors, prior to RC. The appeal of these agents stems from their commendable safety profiles coupled with their pronounced efficacy [36,37]. Consequently, such advancements could profoundly alter the therapeutic trajectory for bladder cancer. Moreover, we may observe a decline in the number of patients with positive LN post-neoadjuvant therapy. Additionally, the survival rates of these patients might align with or even surpass that of patients who opt for immediate RC, independent of the localized tumour stage discerned in the surgical specimens. Also, we are aware that patients included in our study were only treated in two institutions in a single country. To confirm our findings, a larger number of institutions and patients should be included in further studies.

Nevertheless, this study provides a comprehensive analysis elucidating the impact of NAC on the survival of patients with either confirmed negative or positive LNs in RC specimens. A salient feature of our research lies in its innovative findings that delve into the diminished survival rates of patients with <pT2pN+ bladder cancer treated with NAC compared with those who were not. Currently, only one study directly compares the contemporary outcomes after RC in patients with pN+ treated with or without NAC. This particular investigation underscored the potential for a poorer prognosis in patients diagnosed with nodal disease after RC if they were administered NAC [28]. However, our study is the first to indicate that this variation in long-term outcomes is confined to patients who experience NMIBC downstaging, whereas patients manifesting persistent MIBC in conjunction with positive LN resonate closely with those undergoing immediate RC without NAC.

## 5. Conclusions

In conclusion, our study underscores the declining prognosis and OS rates of patients who exhibit persistent nodal disease in RC specimens post-NAC, compared with those opting for immediate surgery. This disparity in outcomes was particularly evident in patients who achieved local non-muscle-invasive downstaging. Given these findings, contemplating adjuvant treatment, coupled with routine follow-up, is crucial for such patients. Future research efforts should aim to categorize patients with <ypT2N+ disease into distinct low- and high-risk groups, based on recurrence and cancer-specific mortality metrics. Such refined stratification could be instrumental in devising personalized treatment pathways tailored for this particular subgroup of patients.

## Figures and Tables

**Table 1:** Baseline patients’ characteristics.

Variable	(y)pN0	(y)pN+	Total	*p* Value
Totals, No.	913	481	1394	
Age, years				0.426
Mean	64.951	65.358		
SD	9.235	8.741		
Gender, No.				0.391
Male	737	379	1116	
Female	176	102	278	
ASA score, No.				<0.001
1	61	8	69	
2	552	281	833	
3	271	182	453	
4	29	9	38	
Clinical T stage, No.				<0.001
cT2	256	39	295	
cT3	501	244	745	
cT4	156	198	354	
Pathological T stage, No.				<0.001
pT0	119	12	131	
pTis/Ta/T1	200	35	235	
pT2	227	41	268	
pT3	204	193	397	
pT4	163	200	363	
Cancer grade, No.				<0.001
Low	95	14	166	
High	818	467	1228	
Chemotherapy regimen, No.				0.092
None	698	379	1077	
MVAC	51	29	80	
ddMVAC	68	21	89	
Gemcitabine-cisplatin	76	48	124	
Gemcitabine-carboplatin	8	2	10	
Gemcitabine-paclitaxel	12	2	14	
Cycles of chemotherapy, No.				
<3	59	44	103	0.005
≥3	156	58	214	

ASA score: American Society of Anaesthesiologists score; ddMVAC: dose-dense methotrexate, vinblastine, doxorubicin, and cisplatin; MVAC: methotrexate, vinblastine, doxorubicin, and cisplatin; SD: standard deviation.

**Table 2:** Multivariable Cox regression analyses for predictors of overall survival stratified by pathologic lymph node status.

All Patients								
	HR	Lower 95%CI	Upper 95%CI	*p*	HR	Lower 95%CI	Upper 95%CI	*p*
	(y)pN0				(y)pN+			
Age, years	1.016	1.005	1.028	0.005	1.017	1.005	1.029	0.007
Gender								
Male	Ref.	Ref.	Ref.		Ref.	Ref.	Ref.	
Female	1.070	0.832	1.376	0.599	1.017	0.792	1.307	0.894
ASA score								
1–2	Ref.	Ref.	Ref.		Ref.	Ref.	Ref.	
3–4	1.244	1.010	1.532	0.040	1.404	1.143	1.725	0.001
Pathological T stage								
≤pT2	Ref.	Ref.	Ref.		Ref.	Ref.	Ref.	
>pT2	2.224	1.790	2.762	<0.001	2.037	1.479	2.806	<0.001
Cancer grade								
Low	Ref.	Ref.	Ref.		Ref.	Ref.	Ref.	
High	1.274	0.837	1.941	0.259	0.885	0.414	1.892	0.752
NAC								
No	Ref.	Ref.	Ref.		Ref.	Ref.	Ref.	
Yes	0.692	0.524	0.915	0.010	0.927	0.713	1.205	0.572
**<(y)pT2**								
	**(y)pN0**				**(y)pN+**			
Age, years	1.036	1.010	1.063	0.007	1.063	0.970	1.164	0.191
Gender								
Male	Ref.	Ref.	Ref.		Ref.	Ref.	Ref.	
Female	1.380	0.796	2.391	0.251	2.599	0.660	10.240	0.172
ASA score								
1–2	Ref.	Ref.	Ref.		Ref.	Ref.	Ref.	
3–4	2.139	1.362	3.359	0.001	5.670	1.781	18.054	0.003
Cancer grade								
Low	Ref.	Ref.	Ref.		Ref.	Ref.	Ref.	
High	1.037	0.635	1.693	0.885	1.672	0.396	7.063	0.484
NAC								
No	Ref.	Ref.	Ref.		Ref.	Ref.	Ref.	
Yes	0.572	0.343	0.951	0.031	3.111	1.249	7.746	0.015
**≥(y)pT2**								
	**(y)pN0**				**(y)pN+**			
Age, years	1.015	1.003	1.028	0.017	1.016	1.003	1.029	0.015
Gender								
Male	Ref.	Ref.	Ref.		Ref.	Ref.	Ref.	
Female	0.994	0.747	1.323	0.967	0.983	0.758	1.274	0.895
ASA score								
1–2	Ref.	Ref.	Ref.		Ref.	Ref.	Ref.	
3–4	1.229	0.970	1.556	0.087	1.329	1.074	1.644	0.009
Cancer grade								
Low	Ref.	Ref.	Ref.		Ref.	Ref.	Ref.	
High	3.484	0.861	14.108	0.080	0.825	0.307	2.220	0.703
NAC								
No	Ref.	Ref.	Ref.		Ref.	Ref.	Ref.	
Yes	0.692	0.493	0.971	0.033	0.758	0.562	1.022	0.069

ASA score: American Society of Anaesthesiologists score; CI: confidence interval; HR: hazard ratio; NAC: neoadjuvant chemotherapy.

## Data Availability

Source data are available at: https://osf.io/prcvt; accessed date: 27 August 2023.

## Supplementary Materials

Table S1: Multivariable Cox regression analyses for predictors of overall survival in the overall cohort. Available at: https://osf.io/xhts6; accessed date: 27 August 2023. Table S2: Propensity score-weighted Multivariable Cox regression analyses for predictors of overall survival stratified by pathologic lymph node status. Available at: https://osf.io/v24s8; accessed date: 27 August 2023.
